# Use of the moving epidemic method (MEM) to assess national surveillance data for respiratory syncytial virus (RSV) in the Netherlands, 2005 to 2017

**DOI:** 10.2807/1560-7917.ES.2019.24.20.1800469

**Published:** 2019-05-16

**Authors:** Laura M Vos, Anne C Teirlinck, José E Lozano, Tomás Vega, Gé A Donker, Andy IM Hoepelman, Louis J Bont, Jan Jelrik Oosterheert, Adam Meijer

**Affiliations:** 1 University Medical Centre Utrecht, Utrecht University, Department of Internal Medicine and Infectious Diseases, Utrecht, the Netherlands; 2 Centre for infectious Disease Control Bilthoven, Centre for Infectious Diseases, Epidemiology and Surveillance, National Institute for Public Health and the Environment (RIVM), Bilthoven, the Netherlands; 3 Dirección General de Salud Pública, Consejería de Sanidad, Valladolid, Spain; 4 NIVEL Primary Care Database – Sentinel Practices, Utrecht, the Netherlands; 5 Wilhelmina Children’s Hospital, Utrecht University, Department of Paediatric Infectious Diseases, Utrecht, the Netherlands; 6 Centre for infectious Disease Control Bilthoven, Centre for Infectious Diseases Research, Diagnostics and laboratory Surveillance, National Institute for Public Health and the Environment (RIVM), Bilthoven, the Netherlands

**Keywords:** RSV, surveillance, moving epidemic method, epidemiology, respiratory syncytial virus, sentinel surveillance, surveillance, the Netherlands, viral infections

## Abstract

**Background:**

To control respiratory syncytial virus (RSV), which causes acute respiratory infections, data and methods to assess its epidemiology are important.

**Aim:**

We sought to describe RSV seasonality, affected age groups and RSV-type distribution over 12 consecutive seasons in the Netherlands, as well as to validate the moving epidemic method (MEM) for monitoring RSV epidemics.

**Methods:**

We used 2005−17 laboratory surveillance data and sentinel data. For RSV seasonality evaluation, epidemic thresholds (i) at 1.2% of the cumulative number of RSV-positive patients per season and (ii) at 20 detections per week (for laboratory data) were employed. We also assessed MEM thresholds.

**Results:**

In laboratory data RSV was reported 25,491 times (no denominator). In sentinel data 5.6% (767/13,577) of specimens tested RSV positive. Over 12 seasons, sentinel data showed percentage increases of RSV positive samples. The average epidemic length was 18.0 weeks (95% confidence intervals (CI):  16.3–19.7) and 16.5 weeks (95% CI: 14.0–18.0) for laboratory and sentinel data, respectively. Epidemics started on average in week 46 (95% CI: 45–48) and 47 (95% CI:  46–49), respectively. The peak was on average in the first week of January in both datasets. MEM showed similar results to the other methods. RSV incidence was highest in youngest (0–1 and >1–2 years) and oldest (>65–75 and > 75 years) age groups, with age distribution remaining stable over time. RSV-type dominance alternated every one or two seasons.

**Conclusions:**

Our findings provide baseline information for immunisation advisory groups. The possibility of employing MEM to monitor RSV epidemics allows prospective, nearly real-time use of surveillance data.

## Introduction

Respiratory syncytial virus (RSV) infection can lead to acute respiratory infections (ARI) and is an important cause of morbidity, hospital admissions and mortality among children and the elderly (aged > 75 years) [1-5]. Respiratory illness due to the virus can also affect both healthy and high-risk adults (e.g. with chronic heart or lung disease) [6]. 

To guide future priority setting and decision making by national policymakers and immunisation advisory groups, widespread national sentinel data can allow to further understand RSV epidemiology and to better define groups at risk of RSV [7]. Reliable epidemiological data can moreover provide a baseline to assess possible future RSV immunisation effects, as one of the candidate vaccines currently in development for RSV might become available within a few years from now [7]. In order to identify potential risk groups for a complicated course of infection, sentinel data [7] are needed to complement the currently available evidence resulting from studies that focus on either adults or children [3], immunocompromised [8], chronically ill persons [9] or hospitalised patients [10,11]. Sentinel data are also relevant for studies modelling RSV epidemiology [4]. In Europe, over the past 15 years, several large surveillance systems have already been deployed, but have mainly focused on children [12-14].

Apart from risk-group identification, reliable epidemiological RSV data may reveal seasonal patterns like the timing, length and peak of the RSV season with importance for awareness, prevention and treatment. Meteorological factors, latitude and co-infection or competition with other respiratory viruses may play an important role in RSV seasonality [14-17]. Although seasonality of RSV at a European level has been evaluated, the available data do not enable to combine seasonal information with RSV-type and important patient characteristics such as age [14]. Furthermore, most European country surveillance systems lack a sufficient number of monitored seasons to properly assess potential evolutions in seasonality [14,18] and during each season, there have been difficulties in determining the intensity of the RSV epidemic.

Indeed, the common use of a certain percentage of RSV positivity among all RSV detections in a season as a threshold to define an epidemic [14,17,18] does not provide insight in the intensity of the epidemic and cannot be employed prospectively to signal the start of an epidemic. For influenza, another viral respiratory disease, another approach, the moving epidemic method (MEM), is widely used to calculate epidemic thresholds [19,20]. This method has the advantage of allowing the calculation of intensity levels and can be used prospectively.

The objectives of this study were to determine whether the MEM is suitable for use in RSV surveillance and to thoroughly evaluate RSV seasonality over 12 consecutive seasons (2005/06 through 2016/17) in the Netherlands by using virological laboratory surveillance data and sentinel general practitioner (GP) surveillance data. For sentinel GP surveillance data, we analysed the occurrence of RSV-A and -B and the distribution of RSV infection in different age groups within the 12 consecutive seasons and assessed whether these distributions changed over time.

## Methods

### Data collection

Dutch national virological laboratory surveillance and sentinel GP surveillance data from week 30 in 2005 through week 29 in 2017 were used, covering 12 consecutive (northern hemisphere) viral respiratory seasons.

#### Virological laboratory surveillance 

Virological laboratory surveillance data consisted of virology diagnostic reports, which are established in the Netherlands since 1964 [21,22]. These data consist of positive virological laboratory diagnostic results of 31 virus species with some distinct (sub)types and serotypes. Some intracellular growing bacteria are also reported. The reports are generated weekly in an online registration system by up to 21 participating Dutch laboratories, which are all members of the Working Group for Clinical Virology of the Dutch Society for Medical Microbiology. The participating laboratories are spread all over the country (Supplementary Figure S1a) and include both hospital laboratories (n = 13) and regional laboratories (n = 8) covering 29–44% of the Dutch population [23]. The data in the weekly reports are based on outcomes of diagnostic tests, either virus isolation, antigen test, PCR or serological test, performed by these laboratories upon request from GPs, clinical departments in hospitals, and outpatient clinics. Apart from the number of RSV diagnoses per week, no further information on individual patients is collected. However, based on an inventory study, we assume that these RSV virological laboratory surveillance data mainly come from very young children, mostly below the age of 6 months [23].

#### Sentinel surveillance – Netherlands institute for health services research primary care database

Sentinel surveillance data in the Netherlands are assembled in the primary care database of the Netherlands institute for health services research (NIVEL) [24]. In the sentinel surveillance, participating GPs collect for viral diagnostics nose and throat swabs from a subset of patients presenting with influenza-like illness (ILI) or another ARI according to Dutch definitions (Supplementary Text S1). The swabs are screened for enterovirus, influenza virus, rhinovirus and RSV at the National Institute for Public Health and the Environment (RIVM), Bilthoven, which is the centre for infectious disease control in the country. The population of the 40 sentinel practices covers ca 0.8% of the Dutch population [25] and is nationally representative for age, sex, regional distribution and population density (Supplementary Figure S1b) [24]. Clinical and epidemiological characteristics from the individual patients are obtained using a standardised questionnaire filled by the GPs. Details on the sampling strategy, e.g. instructions for the GP, are described in Supplementary Text S2a, and on the PCR technique to detect RSV in Supplementary Text S2b *.* Since the duration of RSV shedding in an outpatient setting is reported to be on average 9.8 ± 4.8 days for adults [26] and can be even higher in children (especially of very young age) and immunocompromised patients [27], patient samples were only included in the analysis when the PCR sample was taken within 14 days after start of symptoms. International Organization for Standardization (ISO)-weeks, years and seasons were determined using the day of sampling. Missing values for immune status were imputed using a multiple imputation model including all other clinical variables, further described in Supplementary Text S3.

### Data analysis for seasonality

To describe seasonality of RSV, both virological laboratory and sentinel GP data of 12 consecutive seasons were used. A season was defined from week 30 through week 29 of the following year to capture at least 10 weeks of assumed background noise before and after the epidemic. Characteristics of epidemics in each season were assessed using three methods: (i) epidemic thresholds at 1.2% of the cumulative number of RSV-positive patients per season [14,17], (ii) epidemic thresholds at 20 detections per week (used only for laboratory data) – which is used in daily practice at the RIVM [25] and (iii) epidemic thresholds using MEM [19,28]. For each method we calculated (i) the mean length of the epidemic period, defined as the number of weeks from the first week above the pre-epidemic threshold through the last week above the post-epidemic threshold, (ii) the mean starting and ending week (e.g. timing) of the epidemic period, (iii) the mean timing of the peak week and (iv) the coverage of the epidemic and peak week, defined respectively as the percentages of RSV detections during the determined epidemic period and the peak week relative to all RSV detections during the season (week 30 through 29 of the next year). The RSV epidemic period was defined as the longest period during the season above the threshold, without interruptions for laboratory data and with interruptions of 1 week maximum below or at the threshold for sentinel GP data. The peak week was defined as the first week during the epidemic period with the maximum absolute number of RSV detections per week during that season. Epidemic thresholds and intensity levels were rounded down to the closest integer. All other numbers, including week numbers, were rounded up or down according to the normal rounding rules.

### Data analysis – moving epidemic method

MEM was applied with the Moving Epidemic Method Web Application [29] and absolute detection numbers per week for all 12 seasons in the fixed criterium model using a manually optimised slope parameter. 

We used absolute numbers since the number of reporting sites remained similar and the number of tested samples remained relatively stable over time (Table 1). Furthermore, due to low sample numbers at the beginning and end of the season, relative numbers may be subject to important variations concerning the epidemic and intensity thresholds, which can potentially lead to false alerts.

**Table 1:** Numbers or proportions of samples testing positive for respiratory syncytial virus (RSV) per season (week 30–week 29) obtained from non-sentinel or sentinel surveillance, Netherlands, 2005/06–2016/17 (n = 39,068 tested and positive samples)

Season (week 30–29)	Number of non-sentinel RSV positives^a^ (n)	Number of sentinel RSV tested (n)	Number of sentinel RSV positives (n)	Proportion of sentinel RSV positives/tested (%)	Number of sentinel RSV A (n)	Proportion of sentinel RSV A positives (%)	Proportion of sentinel RSV B positives (%)	Dominant RSV type^b^
2005/06	2,236	516	29	5.6	18	62	38	A
2006/07	1,957	599	23	3.8	6	26	74	B
2007/08	2,160	964	26	2.7	20	77	23	A
2008/09	2,510	1,257	43	3.4	25	58	42	A/B
2009/10	3,103	1,968	99	5.0	44	44	56	A/B
2010/11	2,736	1,290	75	5.8	49	65	35	A
2011/12	1,883	1,092	49	4.5	18	37	63	B
2012/13	2,225	1,258	59	4.7	45	76	24	A
2013/14	1,637	914	71	7.8	31	44	56	A/B
2014/15	1,699	1,306	67	5.1	24	36	64	B
2015/16	1,392	1,335	109	8.2	39	36	64	B
2016/17	1,953	1,078	117	10.9	82	70	30	A
Total	**25,491**	**13,577**	**767**	**5.6**	**401**	**52**	**48**	**NA**

NA: not applicable.

^a^ Denominator for non-sentinel data are unknown, so no percentages could be presented.

^b^ A/B indicates a co-dominance of RSV type A and B.

For both virological laboratory surveillance and sentinel GP data, when a season included a year with a 53rd week in that season it was pulled forward 1 week to avoid gaps in other seasons [30]. 

Manually optimised slope parameters were determined for both datasets separately using a slope range setting from 0.1 to 4.0. Sensitivity and a minimal epidemic percentage of 90% were used to determine the optimum, to be able to detect the start of the epidemic season at an early stage without creating too many false signals. Four researchers with expertise in surveillance (JL, TV, AM and LV) independently performed manual optimisation after which any discrepancies were solved by consensus. After determination of the optimal slope parameters − 1.4 for both datasets − the final analysis was done.

We calculated the mean length, timing and coverage of the epidemic period by calculating pre-and post-epidemic thresholds using the arithmetic mean and its one-sided 95% point confidence interval (CI). We also calculated epidemic intensity levels using the geometric mean and its one sided 40% (medium), 90% (high) and 97.5% (very high) point CI. The validity of MEM epidemic thresholds for detecting the epidemics was calculated using cross validation, reflected as sensitivity, specificity and positive and negative predictive values. All calculations are included in the application.

### Data analysis – age and respiratory syncytial virus type distribution

Distribution of RSV by age was evaluated using sentinel GP data. The mean ages of RSV positive patients vs RSV negative patients were compared using a two-sided independent sample t-test. For clinical applicability, age was not used as continuous variable, but as categorical variable using eight different age groups: 0–1 years, >1–2 years, >2–5 years, >5–15 years, >15–45 years, >45–65 years, >65–75 years, > 75 years. Percentages of RSV positives per age group were compared using a chi-squared test with a two-sided alpha of 0.05. Odds ratios (OR) for RSV positivity were calculated using a logistic regression model using the age group with the lowest proportion RSV positives as reference group. Variability of age distributions among RSV positive patients over the 12 consecutive seasons was assessed visually using spaghetti plots for RSV. The distribution of RSV-A vs RSV-B was calculated per season, after which RSV-A or RSV-B dominance or co-dominance was established using the 60/40 rule used in influenza surveillance [31]. The coincidence between RSV-type distribution and seasonality was assessed visually. All analyses were performed in the Moving Epidemic Method Web Application [29] and IBM SPSS Statistics version 21.

### Ethical statement

According to Dutch legislation, surveillance of ILI/ARI is registered in the Personal Data protection Act Register of the Personal Data Protection Commission. For anonymised studies based on specimens collected through this surveillance no specific ethical approval is needed. Nevertheless, GPs are requested to ask participants (or, for those unable to provide consent, their relatives) informed consent for further anonymised research use of collected specimens and clinical data and register this on the specimens form. None of the patients from whom specimens and data are used in this study objected to further use.

## Results

**Figure 1:**
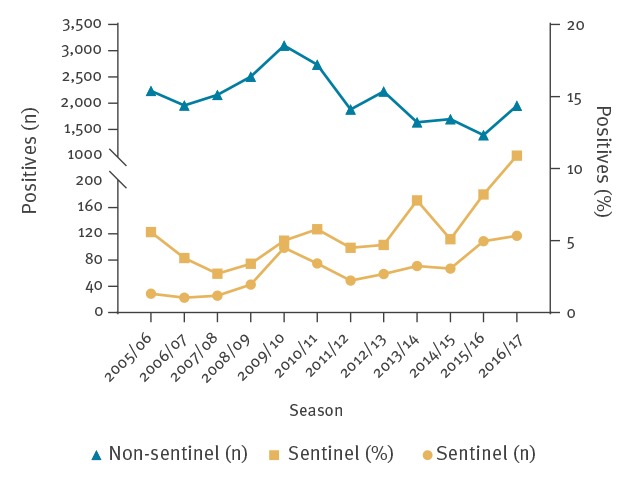
Number and proportions of respiratory syncytial virus positive samples per season (week 30–week 29) obtained from non-sentinel and sentinel surveillance, Netherlands, 2005/06–2016/17 (n = 39,068 samples)

In virological laboratory surveillance data, RSV was reported 25,491 times from week 30 in 2005 (25 July) through week 29 in 2017 (until 24 July). In sentinel GP data, RSV was reported in 767 (5.6%) of 13,577 included patients, of whom 401 (3.0%) were RSV-A and 366 (2.7%) were RSV-B positive. Within sentinel GP data, the percentage of RSV positives per season increased over the 12 seasons (p < 0.001) (Table 1 and Figure 1). The median age of all sentinel GP patients was 36.3 years (interquartile range (IQR): 15.2–54.2 years). RSV positive patients were significantly younger than RSV negative patients with a median age of 11.6 (IQR: 1.5–54.3) years vs 36.8 (IQR: 16.4–54.2) years (p < 0.001). RSV occurred more frequently among immunocompromised patients (9.4%; 14/149) than among immunocompetent patients (5.6%; 753/13,428) (p = 0.046). Among ILI patients 5.1% (365/7,160) of patients was RSV positive, whereas among other ARI patients 6.3% (402/6,417) was RSV positive (p = 0.003).

### Seasonality and moving epidemic method validation – virological laboratory surveillance data

**Figure 2:**
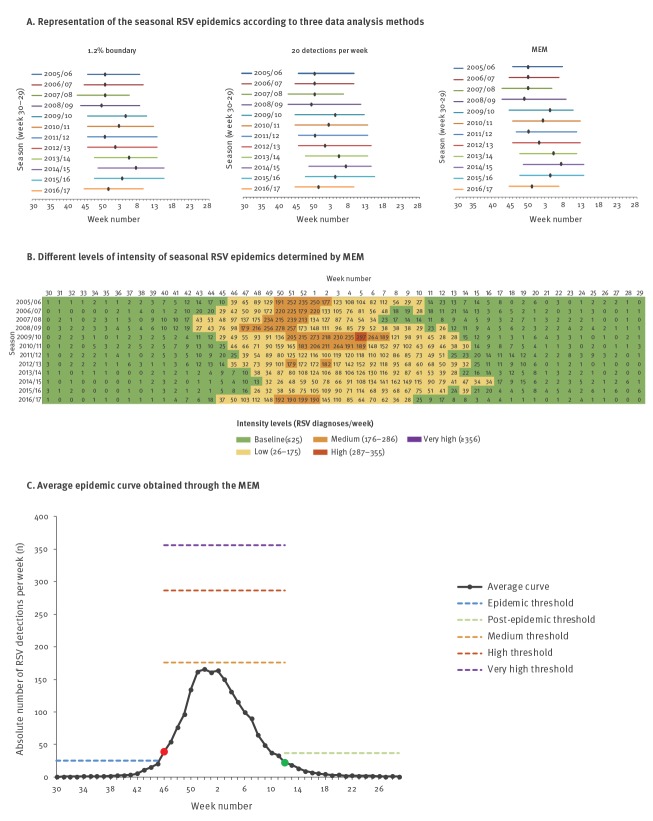
Laboratory surveillance data-based representation of (A) seasonal RSV epidemics, (B) seasonal epidemic intensity levels determined by MEM and (C) average epidemic MEM curve, Netherlands, 2005/06–2016/17 (n = 25,491 RSV positive samples)

MEM: moving epidemic method; RSV: respiratory syncytial virus.

On average, RSV was detected 2,125 (95% CI: 1,701–2,549) times per season (week 30–29). The average MEM pre- and post-epidemic thresholds were 25 and 37 RSV diagnoses per week, respectively. Figure 2 shows graphs of RSV epidemic periods, MEM intensity levels and the average epidemic MEM curve for virological laboratory surveillance data. The epidemic period (Figure 2A) was sometimes shorter than the period of low intensity near the end of the epidemic (Figure 2B), since the post-epidemic threshold was higher than the low intensity threshold. For example, for 2005/06 MEM determines the RSV season to end in week 9, but the low level intensity continues until week 10.

A. Length and timing of the epidemic period per season (coloured horizontal lines) and timing of the peak (black dots) obtained using three different methods: (i) 1.2% of total number of RSV positives per season, (ii) 20 detections per week and (iii) the MEM method with an optimised slope parameter of 1.4.

B. Heat map showing the different levels of intensity given by the MEM method for each season: baseline (≤25 RSV diagnoses/week; green), low (26−175 RSV diagnoses/week; yellow), medium (176−286 RSV diagnoses/week; orange), high (287−355 RSV diagnoses/week; red) and very high (≥356 RSV diagnoses/week; purple).

C. Average epidemic curve over the overall study period given by the MEM method (MEM web application) with start (first week above pre-epidemic threshold, indicated with a red dot) and end (first week below post-epidemic threshold, indicated with a green dot) of the epidemic period and the three different intensity thresholds (medium, high, very high).

Note that for 2010 and 2016 week 1 is actually week 53 of 2009 and 2015 respectively, and week 2 actually is week 1 of 2010 and 2016 respectively.

Using MEM, the average epidemic length was 18.0 weeks (95% CI: 16.3–19.7), covering 91.5% (95% CI: 90.2–92.2%) of all RSV diagnoses during the season. Using the predefined threshold of 1.2%, the average epidemic period lasts 18.8 weeks (95% CI: 17.8–19.9) and covered 92.4% (95% CI:  91.5–93.2%) of all RSV diagnoses. Using the threshold of 20 detections per week, the average season lasted 19.8 weeks (95% CI: 18.7–20.8) and covered 93.3% (95% CI: 92.2–94.3) of all RSV diagnoses. 

The epidemics started on average in week 46 (95% CI: 45–48) using MEM, also in week 46 (95% CI: 45–47) using the 1.2% method and in week 45 (95% CI: 44–46) using the 20 detections per week method. 

Both the timing of epidemic periods and the timing of the peak weeks followed an amplitude-like pattern, which was most pronounced with MEM (Figure 2A). The timing of the peak was on average in week 1 (95% CI: 51–3) for all three methods covering 9.7% of all RSV diagnoses. 

Using MEM, the sensitivity of the MEM thresholds for the detection of an epidemic was 88.3%, the specificity 92.5%, the positive predictive value (PPV) 85.1% and the negative predictive value (NPV) 94.2%.**

### Seasonality and moving epidemic method validation – sentinel general practitioner data

**Figure 3:**
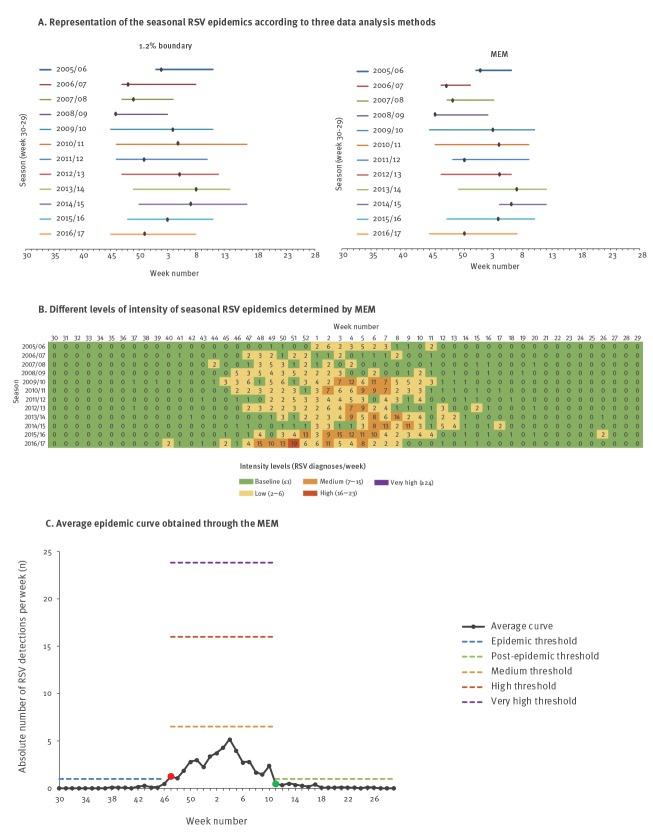
Sentinel general practitioner data-based representation of (A) seasonal RSV epidemics, (B) seasonal epidemic intensity levels determined by MEM and (C) average epidemic MEM curve, Netherlands, 2005/06–2016/17 (n = 767 RSV positive samples)

MEM: moving epidemic method; RSV: respiratory syncytial virus.

On average, RSV was detected 64 (95% CI: 46–82) times per season (week 30–29). Both the average pre-and post-epidemic threshold was one RSV diagnosis per week. Figure 3 shows graphs of RSV epidemic periods, MEM intensity levels and the average epidemic MEM curve for sentinel GP data. 

A. Length and timing of the epidemic period per season (coloured horizontal lines) and the timing of the peak (black dots) obtained using two different methods: (i) 1.2% of total number of RSV positives per season and (ii) the MEM method with an optimised slope parameter of 1.4.

B. Heat map showing the five different levels of intensity given by the MEM method for each season: baseline (≤1 RSV diagnosis/week; green), low (2−6 RSV diagnoses/week; yellow), medium (7−15 RSV diagnoses/week; orange), high (16−23 RSV diagnoses/week; red) and very high (≥24 RSV diagnoses/week; purple).

C. Average epidemic curve over the overall study period given by the MEM method (MEM web application) with start (first week above pre-epidemic threshold, indicated with a red dot) and end (first week below post-epidemic threshold, indicated with a green dot) of the epidemic period and the three different intensity thresholds (medium, high, very high).

Note that for 2010 and 2016 week 1 is actually week 53 of 2009 and 2015 respectively, and week 2 actually is week 1 of 2010 and 2016 respectively.

Using MEM, the average epidemic length was 16.5 weeks (95% CI: 14.0–18.0), covering 92.9% (95% CI: 90.2–95.3%) of all RSV detections during the season. Using the predefined threshold of 1.2%, the average epidemic period typically lasted 16.0 weeks (95%CI: 13.6–18.4) and covered 91.5% (95% CI: 87.7–95.4%) of all RSV detections. 

The epidemic started on average in week 47 (95% CI: 46–49) using MEM and in week 47 (95% CI: 46–48) using the 1.2% method and, similar to virological laboratory surveillance data, followed an amplitude-like pattern (Figure 3A). 

The mean timing of the peak was in week 1 (95% CI: 50–4) using both methods, covering on average 15.0% of all RSV detections during the season. 

Using MEM, the sensitivity of the thresholds for the detection of an epidemic was 75.1%, the specificity 95.6%, the PPV 87.0% and the NPV 90.8%.

### Age and respiratory syncytial virus-type distribution

**Figure 4:**
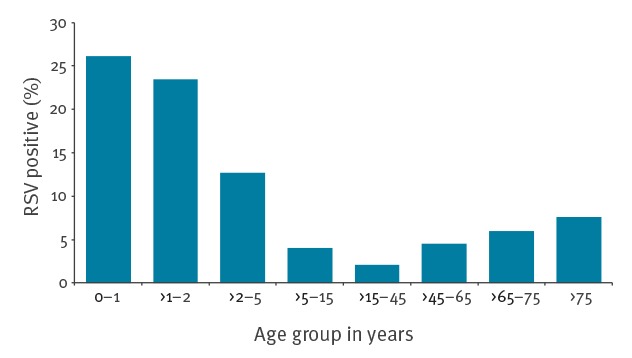
Distribution of respiratory syncytial virus positive patients according to age group, sentinel general practitioner surveillance, Netherlands, 2005/06–2016/17 (n = 767 patients)

For applicability and clear interpretation, age was divided into eight age groups (sentinel data). RSV incidence was highest in the age group 0–1 years (26.2%) and lowest in age group >15–45 years (2.1%) (Figure 4). Taking the >15–45 year olds as reference group, the ORs for RSV positivity for the other age categories were 16.7 (95% CI: 12.7–22.1) for 0–1 years, 14.5 (95% CI: 10.7–19.5) for >1–2 years, 6.9 (95% CI: 5.2–9.1) for >2–5 years, 2.0 (95% CI: 1.4–2.7) for >5–15 years, 2.2 (95% CI: 1.7–2.9) for >45–65 years, 3.0 (95% CI: 2.2–4.1) for >65–75 years and 3.9 (95% CI: 2.7–5.6) for patients > 75 years. 

RSV: respiratory syncytial virus.

**Figure 5:**
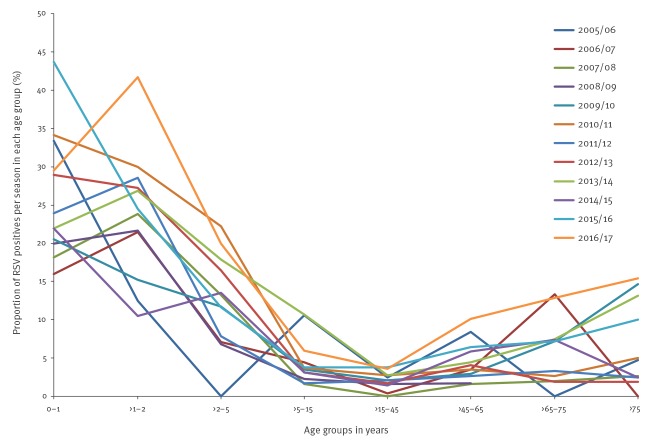
Distribution of patients with respiratory syncytial virus infections in 12 consecutive seasons, according to age group, sentinel general practitioner data, Netherlands, 2005/06–2016/17 (n = 767 patients)

The distribution of RSV positive patients over the eight age groups showed little variation over time, with highest RSV percentages for the ages < 2 years and lowest RSV percentages in age group of >15–45 years (Figure 5). 

RSV: respiratory syncytial virus.

Applying the 60/40 rule for determination of the predominant virus type, RSV-A was dominant in five seasons and RSV-B in four seasons with an alternating pattern every one or two seasons (Table 1). No coincidence between RSV-type dominancy and the amplitude-like pattern timing in start and peak of the RSV epidemic period was observed (Figures 2A, 2B, 3A and 3B).

## Discussion

Our study provides a detailed overview of seasonal patterns in RSV epidemiology in the Netherlands over a period of 12 years. We found RSV in 5.6% of patients presenting with ARI/ILI at the GP, with a significant increase in the percentage of RSV positives over the past 12 years. Although the instructions for inclusion in the sentinel surveillance of NIVEL primary care database (GP data) have remained the same over these 12 years, this increase might still have to do with increasing RSV awareness, resulting in patient samples being taken more selectively at the GPs as well as more selective GP consulting of patients with ARI/ILI [32]; alternatively, it could be due to increased RSV virulence. We rule out that an improved diagnostic sensitivity caused this increase, since all primers and probes of the real-time PCR-methods remained similar over the years (Supplementary Text S2). The RSV epidemic period is quite uniform in length and follows an amplitude-like pattern in the timing of its start. The RSV epidemic period also has a well-defined epidemic peak of 1 week. Virological surveillance data showed a slightly earlier start and longer epidemic period as compared with sentinel GP data, which might be explained by a more vulnerable included patient population and a higher coverage rate in the Netherlands. RSV-type dominance alternates every one or two seasons. Over time, there is only little, negligible, variation in the age distribution of RSV.

To our knowledge, this is the first study that used MEM for RSV, which is so far only widely used in influenza research [19,20]. As compared with two commonly used other methods and with the results of a previous study on a European level that used the 1.2% method [14], MEM gave similar results and could accurately detect the RSV epidemic, especially with virological laboratory surveillance data. In contrast to the 1.2% method that needs the cumulative number detections in a season as denominator [14], MEM has the advantage that it does not need a denominator and can therefore be applied to both virological laboratory surveillance data with absolute numbers of detections and sentinel GP data. The other two methods can either not be applied in sentinel data because the number of detections per week is too low for the 20 detections per week method or face difficulties due to imprecision with percentages with small numbers in sentinel data (1.2% method). In addition, MEM can easily be replicated in other surveillance systems, quantifies the intensity of the RSV epidemic and can be applied to the most recently available epidemic trends. Furthermore, and maybe most importantly, MEM can be used prospectively to set thresholds for the coming season, whereas thresholds based on percentages of the total number of RSV detections within a season can only be used retrospectively. Prospectively determined thresholds and intensity levels may help surveillance authorities to increase awareness in both primary and secondary care settings for the coming season, potentially leading to more focused diagnostics and therapy during the epidemic period. 

As MEM largely depends on the type of data and MEM settings that are used, transparency in the settings and data handling is essential and should be chosen based on sound argumentation [20]. Sensitivity and specificity should be well balanced to minimise false starts of the epidemic due to the regular noise at the beginning of the epidemic period on the one hand and to include a high percentage of all detections within the epidemic period on the other hand. Our usage of expert opinion to optimise the settings of MEM appeared to be a valuable and transparent approach in a situation where MEM is first used for other pathogens or other epidemic patterns.

Our data underline the need for RSV awareness in elderly patients. We found that patients > 65 years old have a threefold and patients > 75 years a 3.9-fold increased risk for RSV infections as compared with adults between >15 and 45 years old, regardless of any underlying diseases or immune status [6]. Even though the selection of age groups largely determines the RSV incidence rates per age group and may therefore cause artificial results, our findings are in line with previous reports that show increased RSV incidence in elderly [33]. According to former studies, RSV does not only have a high incidence but also a high disease burden in elderly patients, with hospitalisation and mortality rates similar to influenza virus infection [34] and outbreaks in nursing homes with fatality rates of 20% (range: 2–20%) [35]. Awareness for this frequent and virulent pathogen is therefore not only needed in a hospital setting, but also in primary care and nursing homes.

The difference between RSV-A and RSV-B originates from structural differences in the G-gene of the virus encoding the important surface G-protein, responsible for attachment to the epithelial cell of the host [36]. The observed alternating pattern in RSV-type predominance is explained by reductions in susceptibility and interactions between antigenic variations in the virus and transmission dynamics [37]. This consistent pattern of changing predominance has important implications for RSV vaccines currently under development [38], and thus need coverage for both RSV-types, although there may be cross-neutralisation of vaccines that target largely conserved genomic regions in the F-protein that is responsible for fusion of the virus with host cell membranes, a requirement for productive infection. In addition, as the debate on clinical impact of RSV-types is still ongoing, it is important to know and understand their seasonal patterns [39,40].

A limitation of the virological laboratory surveillance data is the absence of a denominator, e.g. the number of tested patients, the criteria used for testing, and any clinical data from the included patients. From a previous study on data from 2001 to 2008 we assumed these data were mainly from children less than 6 months of age [23]. However, absence of clinical data limits possibilities to evaluate potential changes since 2008 in the age distribution of RSV-positive patients included in the virological laboratory surveillance data, which might have changed as a result of increasing awareness of the contribution of RSV to respiratory illness in adults and in particular in the most elderly. Nevertheless, by using both sentinel GP and virological laboratory surveillance data and three different methods to assess seasonality we attempted to strengthen the precision of our analysis, resulting in interchangeable seasonality patterns. 

## Conclusion

The current study introduced and validated MEM for characterising RSV epidemics [19,41]. This method has great potential in both clinical practice and surveillance as it enables to determine thresholds based on historical data and to monitor prospectively, nearly in real-time, the start, duration, timing and intensity of an upcoming RSV epidemic [20]. Virological and sentinel surveillance data analyses conducted as part of this study indicate that in the Netherlands RSV infections follow a fairly uniform pattern within the general population in terms of length of the epidemic season and distribution among age groups, with the highest incidence in children < 5 years old and elderly aged > 65 years. Since 2005, the percentage of RSV positives among ILI/ARI patients within sentinel GP surveillance has increased. The timing of the epidemic RSV period has an amplitude-like pattern, which is uncorrelated with the RSV-type dominance that alternates every 1–2 seasons. The findings in this report potentially allow better comparisons of RSV epidemics between years, regions and countries. They also provide an epidemiological base for RSV awareness, which in turn could lead to enhanced detections of infections in both primary and secondary care (particularly for infants and the elderly) and to adequate treatment of the RSV patients identified (e.g. with antivirals such as ribarivin and passive immunisation with palivizumab in severely ill patients and premature infants, respectively). The results can be helpful for national policymakers and immunisation advisory groups to guide priority setting and provide baseline data for assessing any future vaccination programmes. 

## Supplementary Data

356000 Supplementary Material
