# Supplementary material for: Use of the moving epidemic method (MEM) to assess national surveillance data for respiratory syncytial virus (RSV) in the Netherlands, 2005 to 2017
Source: Euro Surveill. 2019 May 16;24(20):1800469. doi: 10.2807/1560-7917.ES.2019.24.20.1800469 (PMC6530251; doi:10.2807/1560-7917.ES.2019.24.20.1800469)
Supplement: Supplementary Material [file 1800469_Vos_SupplementaryMaterial.pdf]

Supplementary Figure S1. ***A.*** *Locations of laboratories providing information for virological weekly reports in the Netherlands (in blue)*[1]*.* ***B.*** *Locations of the 40 sentinel practices participating in 2016 in the national sentinel surveillance network (NIVEL Primary Care Database) in the Netherlands (in white)*[2]*.*

This supplementary material is hosted by Eurosurveillance as supporting information alongside the article "National surveillance data for Respiratory Syncytial Virus (RSV) from 2005 through 2017 in The Netherlands and the introduction of the Moving Epidemic Method (MEM)" on behalf of the authors who remain responsible for the accuracy and appropriateness of the content. The same standards for ethics, copyright, attributions and permissions as for the article apply. Eurosurveillance is not responsible for the maintenance of any links or email addresses provided therein.

A **B**

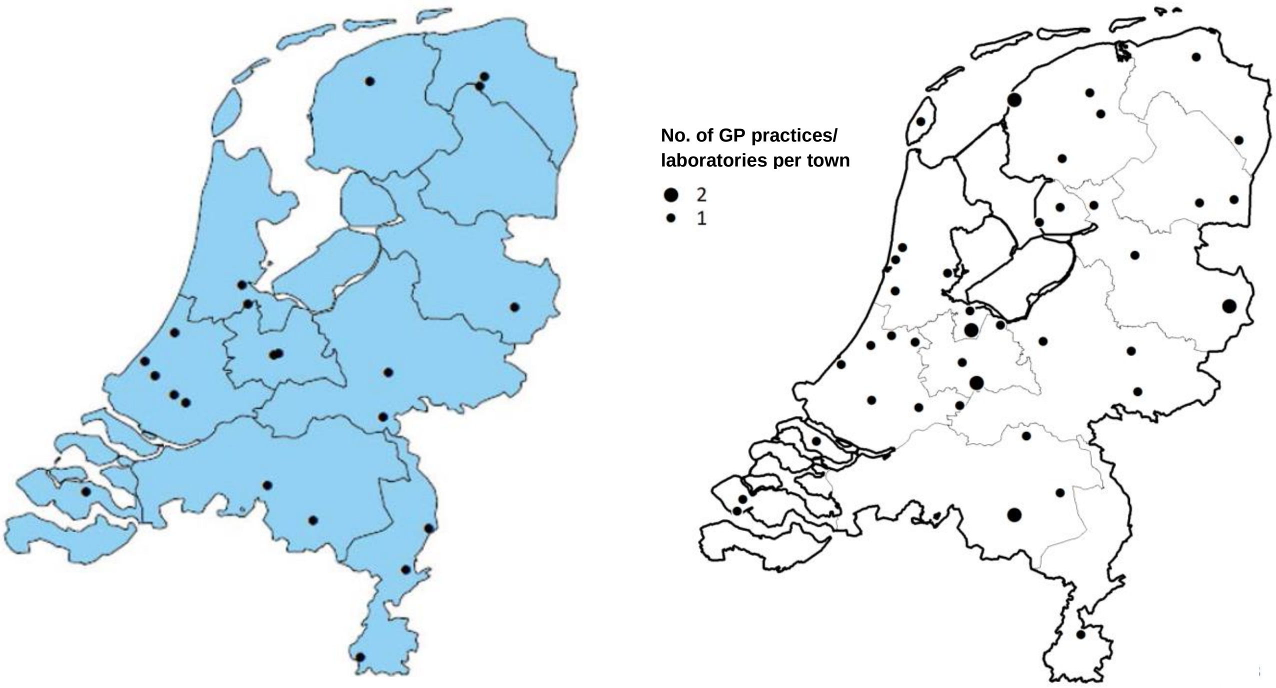

1. Bijkerk P, Gier B de, Nijsten DRE, Duijster JW, Soetens LC, Hahné SJM, et al. State of Infectious Diseases in the Netherlands. RIVM Report 2016:46.

2. G. Donker. [Nivel Zorgregistraties eerste lijn – Peilstations 2017]. Nivel 2018:5.

Supplementary Text S1. *Definitions of ARI and ILI according to Dutch sentinel surveillance.*

This supplementary material is hosted by Eurosurveillance as supporting information alongside the article "National surveillance data for Respiratory Syncytial Virus (RSV) from 2005 through 2017 in The Netherlands and the introduction of the Moving Epidemic Method (MEM)" on behalf of the authors who remain responsible for the accuracy and appropriateness of the content. The same standards for ethics, copyright, attributions and permissions as for the article apply. Eurosurveillance is not responsible for the maintenance of any links or email addresses provided therein.

ARI is defined as a clinical diagnosis of acute upper respiratory infection, acute/chronic sinusitis, acute laryngitis/tracheitis, acute bronchitis/bronchiolitis or influenza[1]. ILI is a subgroup of ARI and is defined as sudden onset of symptoms, fever ≥38^{0}C and a combination of at least one of the symptoms cough, rhinorrhoea, sore throat, frontal headache, retrosternal pair, or myalgia[2].

1. Teirlinck AC, Van Asten L, Brandsema PS, Dijkstra F, Donker GA, Van Gageldonk-Lafeber AB, et al. Annual report Surveillance of Influenza and other respiratory infections in the Netherlands: winter 2016/2017. RIVM Report 2017:117.

2. Pel J. [Proefonderzoek naar de frequentie en de etiologie van griepachtige ziekten in de winter 1963-1964.] Huisarts en Wetenschap 1965;8:321.

Supplementary Text S2. *Developments and changes in sentinel surveillance system between week 30 2005 and week 29 2017.*

This supplementary material is hosted by Eurosurveillance as supporting information alongside the article "National surveillance data for Respiratory Syncytial Virus (RSV) from 2005 through 2017 in The Netherlands and the introduction of the Moving Epidemic Method (MEM)" on behalf of the authors who remain responsible for the accuracy and appropriateness of the content. The same standards for ethics, copyright, attributions and permissions as for the article apply. Eurosurveillance is not responsible for the maintenance of any links or email addresses provided therein.

A. Until the 2014/2015 season, the GP sentinel practices were requested to take specimens of at least two ILI patients per week, of which one patient should be a child below the age of ten years. If no ILI patients were encountered or willing to participate, the GPs were requested to take specimens from ARI patients. Since the 2015/2016 season, the instructions for the GPs to swab ILI patients have changed in order to harmonize better in international collaboration and to obtain specimens and correlated data as systematically as possible. The instructions are since then as follows: 1) swab the first two ILI patients encountered on Monday through Wednesday; 2) when on Monday through Wednesday no ILI patients younger than 65 years are encountered, than swab on Thursday through Sunday the first two ILI patients or ARI patients encountered who are younger than 65 years of age; 3) swab all patients of 65 years and older with an ILI or ARI throughout the week.

B. Until November 2005, conventional polymerase chain reaction (PCR) using agarose gels for detection of PCR amplificates was used to identify RSV, by which no distinction between RSV type A and RSV type B was made. In November 2005 the conventional PCR was replaced by real-time reverse transcription PCR (RT-PCR) using a Roche LightCycler (LC) 2.0 thermal cycler and Taqman® masterkit and newly designed primers and fluorescent labeled probes, which enabled simultaneous detection and typing of RSV type A and B and has higher sensitivity. In the years that followed, there were some minor changes with ignorable effect on sensitivity: in January 2007 the LC 2.0 machine was replaced by a Roche LC 480 Thermal Cycler; in 2010 the Taqman® EZ one-step RT-PCR kit was introduced without any change in primers and probes and in February 2013 the TaqMan® Fast Virus 1-Step Master Mix was introduced, again without changes in primers and probes. The real time RT-PCR assays always had 100% correct score in annual External Quality Assessment studies conducted by Quality Control for Molecular Diagnostics (QCMD), Glasgow, Scotland, UK, a requirement for the RIVM laboratory being accredited according to the ISO 15189 norm.

Supplementary Text S3. *Multiple imputation model.*

This supplementary material is hosted by Eurosurveillance as supporting information alongside the article "National surveillance data for Respiratory Syncytial Virus (RSV) from 2005 through 2017 in The Netherlands and the introduction of the Moving Epidemic Method (MEM)" on behalf of the authors who remain responsible for the accuracy and appropriateness of the content. The same standards for ethics, copyright, attributions and permissions as for the article apply. Eurosurveillance is not responsible for the maintenance of any links or email addresses provided therein.

Multiple imputations were performed to account for missing values using standard settings of SPSS, creating 5 imputation sets. The following variables were incorporated in the imputation model: region of the Netherlands (North, South, East, West) (no missings), gender (64 missings), immune status (2838 missings), age (4 missings), influenza vaccination status (144 missings), type of swab - throat and/or nose swab - (709 missings) and respiratory allergy status (2775 missings). For all variables with missing values, missings were imputed. Pooled data from the 5 imputed datasets were used for further analyses.
